# Efficacy of a Web-Based Guided Recommendation Service for a Curated List of Readily Available Mental Health and Well-Being Mobile Apps for Young People: Randomized Controlled Trial

**DOI:** 10.2196/jmir.6775

**Published:** 2017-05-12

**Authors:** Niranjan Bidargaddi, Peter Musiat, Megan Winsall, Gillian Vogl, Victoria Blake, Stephen Quinn, Simone Orlowski, Gaston Antezana, Geoffrey Schrader

**Affiliations:** ^1^ Digital Psychiatry & Personal Health Informatics Group School of Medicine Flinders University Clovelly Park Australia; ^2^ Young and Well Cooperative Research Centre Melbourne Australia; ^3^ Section of Eating Disorders Department of Psychological Medicine, Institute of Psychiatry, Psychology and Neuroscience King’s College London London United Kingdom; ^4^ ReachOut.com Sydney Australia; ^5^ School of Health Sciences Department of Statistics Data Science and Epidemiology Swinburne University Melbourne Australia

**Keywords:** well-being, mental health, young people, online intervention, apps, engagement

## Abstract

**Background:**

Mental disorders are highly prevalent for the people who are aged between 16 and 25 years and can permanently disrupt the development of these individuals. Easily available mobile health (mHealth) apps for mobile phones have great potential for the prevention and early intervention of mental disorders in young adults, but interventions are required that can help individuals to both identify high-quality mobile apps and use them to change health and lifestyle behavior.

**Objectives:**

The study aimed to assess the efficacy of a Web-based self-guided app recommendation service (“The Toolbox”) in improving the well-being of young Australians aged between 16 and 25 years. The intervention was developed in collaboration with young adults and consists of a curated list of 46 readily available health and well-being apps, assessed and rated by professionals and young people. Participants are guided by an interactive quiz and subsequently receive recommendations for particular apps to download and use based on their personal goals.

**Methods:**

The study was a waitlist, parallel-arm, randomized controlled trial. Our primary outcome measure was change in well-being as measured by the Mental Health Continuum-Short Form (MHC-SF). We also employed ecological momentary assessments (EMAs) to track mood, energy, rest, and sleep. Participants were recruited from the general Australian population, via several Web-based and community strategies. The study was conducted through a Web-based platform consisting of a landing Web page and capabilities to administer study measures at different time points. Web-based measurements were self-assessed at baseline and 4 weeks, and EMAs were collected repeatedly at regular weekly intervals or ad hoc when participants interacted with the study platform. Primary outcomes were analyzed using linear mixed-models and intention-to-treat (ITT) analysis.

**Results:**

A total of 387 participants completed baseline scores and were randomized into the trial. Results demonstrated no significant effect of “The Toolbox” intervention on participant well-being at 4 weeks compared with the control group (*P*=.66). There were also no significant differences between the intervention and control groups at 4 weeks on any of the subscales of the MHC-SF (psychological: *P*=.95, social: *P*=.42, emotional: *P*=.95). Repeat engagement with the study platform resulted in a significant difference in mood, energy, rest, and sleep trajectories between intervention and control groups as measured by EMAs (*P*<.01).

**Conclusions:**

This was the first study to assess the effectiveness of a Web-based well-being intervention in a sample of young adults. The design of the intervention utilized expert rating of existing apps and end-user codesign approaches resulting in an app recommendation service. Our finding suggests that recommended readily available mental health and well-being apps may not lead to improvements in the well-being of a nonclinical sample of young people, but might halt a decline in mood, energy, rest, and sleep.

**Trial Registration:**

Australian New Zealand Clinical Trials Registry (ANZCTR): ACTRN12614000710628; https://www.anzctr.org.au/Trial/Registration/TrialReview.aspx?id=366145 (Archived by WebCite at http://www.webcitation.org/ 6pWDsnKme)

## Introduction

### Background

In Australia, clinical mental disorders are highly prevalent among young adults aged between 16 and 25 years. Approximately one in four suffer from at least one diagnosable mental disorder in the past 12 months [1], and mental disorders account for a quarter of the burden of disease in this age group [2]. With similar prevalence rates globally (eg, in Europe, Africa, the United States, and Asia) adolescent mental health is an international public health challenge [3]. Adolescence is a crucial developmental stage for the individual and disruption to mental health during this stage can have far-reaching effects, and whose full personal and socioeconomic impact often only becomes apparent at a later stage in life [3]. Thus, effective, engaging, and easy to disseminate strategies that reduce the multiplicative impact of these risk factors within young people are needed.

### mHealth Apps

Although technology mediated mental health interventions have frequently been praised for their potential and ease of access, previous research into one-size-fits-all intervention has demonstrated that these interventions have limited appeal and that they have failed to gain traction within health care [4]. It is possible that this limitation can be overcome by delivering interventions using mediums and resources young people are already interacting with and that are tailored to their circumstances. Mobile health (mHealth) apps have great potential for the prevention and early intervention of many physical and mental health problems. To date, there are approximately 165,000 health apps available for Android and iOS mobile phones and tablet devices, approximately 10% of which are mobile apps for mental health problems [5]. However, it has been shown that many easily available mHealth apps are of dubious quality and do not follow evidence-based principles [5]. For example, research into currently available apps for individuals with bipolar disorder found that the majority were not developed in line with best practice clinical guidelines or self-management principles currently used in the treatment of bipolar disorder. Most apps also did not contain source citations or privacy policies, making it difficult for users to assess app quality [6]. An evaluation of mobile apps for mindfulness highlighted that many apps often claim to be for a particular purpose or provide a particular intervention when they in fact do not [7]. Those mobile mental health apps that are based on evidence-based principles and have demonstrated efficacy often have been developed as part of research studies and are not available publically [8]. This suggests that there is a gap between evidence-based research and readily available existing mHealth interventions in the open market. Therefore, to utilize the public health potential of these existing mHealth apps, it is important to identify those apps of high quality and guide individuals in finding interventions that meet their need and likely work.

### Rating Apps

With regard to identifying high quality apps, most people use app store ratings as a marker for app quality, as indicated by the correlation between app user ratings and their popularity on the marketplace [9]. However, these ratings mainly reflect subjective experiences from a usability and aesthetics perspective, and not whether the apps are designed with appropriate strategies necessary to improve health outcomes [10]. Obtaining more objective evaluations of mobile apps with regard to their quality is hampered by the fact that even with regard to simple criteria, such as the degree of personalization, the funding source of related research, or data import and export capabilities, interrater reliability is poor [11]. In an attempt to overcome the limitations of user ratings for mobile apps, Stoyanov et al [10] created the mobile app rating scale (MARS), a questionnaire to assess the quality of health apps on the domains engagement, functionality, aesthetics, information, and subjective quality. Whereas this scale is a more objective marker of app quality than app store ratings, it is also resource intensive and requires thorough assessment. As a result, application of this approach on a large scale is still in its infancy due to the large volume of mHealth apps.

With regard to identifying effective apps, research suggests that theory-driven health interventions, that is, interventions employing evidence-based behavior change techniques (BCTs) are more effective than interventions that are not theory-driven [12]. The implementation of such strategies within mobile apps is influenced by how users interact with mobile apps [13]. Mobile phone apps are generally optimized to the way people engage with such phones and as such tend to implement only some strategies with functionalities that are brief and easy to use. As a result, a single app alone is unlikely to contain all necessary strategies for mental health, and furthermore identical strategies might be implemented in different apps with varying aesthetics and ease of use. Thus, characterizing strategies implemented within an app will be a crucial step to identify effective apps. To ensure that ethical values in health care are met, this characterization process could be facilitated by clinicians or researchers by reviewing the scientific literature, searching apps stores, reviewing app descriptions and reviews, and piloting the app themselves [14]. However, it is important to recognize that individuals are less likely to engage with interventions that implement effective strategies but poor aesthetics and usability, thus resulting in ineffective outcomes [15,16].

One way of overcoming these challenges is to create a repository of high-quality mHealth apps and guide users in the process of identifying effective and engaging ones. The “Beacon” website is one such resource developed in Australia that categorizes, reviews, and rates websites and mobile apps for mental and physical disorders [17]. Recent findings suggest participants are willing to use several apps when they are recommended a range of custom-selected apps with different behavioral strategies [13]. The challenges of this approach have been highlighted in the UK’s National Health Service’s attempt of creating a curated app repository for patients with chronic health conditions. Many of the apps were found to transmit sensitive data without the knowledge of the user [18] or provide clinically questionable advice [19] that resulted in the app library to be closed. In addition, the majority of apps are generally identified and downloaded by users directly from apps stores and the keywords people use when searching for specific health apps do not necessarily yield the most appropriate or effective app [20]. Instead they might be reflective of these words appearing in place like app name, text used in description of the apps combined with the user rated popularity of the apps, none of which alone are markers of quality. Developing a nuanced understanding of search patterns for mental health and well-being problems will be crucial to facilitate access to apps of high quality.

### Impact Assessment

Methods to measure the impact of app usage may require different approaches, given the way people tend to interact with apps, usually for short periods of time, on a regular or irregular basis. An alternative to traditional questionnaire-based measures of mental health and well-being, ecological momentary assessment (EMA) [21] may be a suitable means of detecting short-term changes with regard to parameters, such as mood or sleep on a day-to-day basis.

In this paper, we report on findings from a waitlist randomized controlled trial (ACTRN 12614000710628) [22] which was designed to test the efficacy of a guided recommendation service for readily available mobile mental health apps for young people aged 16-25 years. Whereas our primary outcome measure was the well-validated Mental Health Continuum-Short Form (MHC-SF), we employed ecological momentary assessments to track mood, sleep, and energy.

## Methods

### Overview

We conducted a Web-based parallel-arm randomized controlled trial comparing “The Toolbox,” a guided app recommendation service, to a waiting list control group. Web-based measurements were assessed at baseline and 4 weeks, and ecological momentary assessments were collected repeatedly at regular weekly intervals or ad hoc when participants interacted with the study platform. Details of study design, intervention and control conditions, outcome measures, and sample sizes are reported extensively in the previously published study protocol [22]. A brief overview of the study is outlined in the following section. The study received ethical approval by the Social and Behavioural Research Ethics Committee of Flinders University (registration number 64780) and is registered in the Australian New Zealand Clinical Trials Registry (ACTRN: 12614000710628). It also gained ethical approval for recruitment by the Department of Education and Child Development of South Australia (DECD CS/14/511-23).

### Recruitment

Participants were recruited from the general young adult (16-25 years) population across Australia, with access to a computer or mobile phone and the Web. Preexisting mental health conditions were not considered as exclusion conditions for this study. Several Web-based and community strategies, either paid or unpaid, were utilized for recruitment. The recruitment message was formulated around overall well-being and health (and not on illnesses): Examples of such messages included:

Want to improve your energy and fitness? Find out what your wellbeing looks like and use apps to achieve your goals.

Better health & fitness: Monitor your wellbeing, set goals, & access health & fitness apps.

Summer fun taking its toll? Track your wellbeing & download apps for mind+body.

Web-based paid advertisements were placed on Facebook, Twitter, YouTube, and Google AdWords from November 19, 2014 to March 12, 2015. The keywords for the advertisements were defined in collaboration with a reference group representing the target population to ensure their validity and relevance. Examples of keywords included fitness, stress, relationships, balance, and goals. A total of 12 advertisements were placed across the previously mentioned platforms, with an average duration of 21 days per advertisement. The paid strategy also included recruitment through a recruitment agency for clinical trials. The agency referred individuals in the target demographic to the study website over a period of 2 months (July 8, 2015 to September 2, 2015). In addition to paid advertisements, links to the study site were provided to 39 organizations and educational institutions frequently visited by young people from different backgrounds in Australia (most notably the partner organizations of the Young and Well Cooperative Research Centre) to integrate into their websites and promote via their social media channels (Facebook, Twitter). Community-based organizations such as schools, universities, sporting clubs, and local councils in one rural region of South Australia were approached and asked to help promote the study. Promotional packages comprising a video, information, and instructions on how to access the Online Wellbeing Centre (OWC) were distributed to 32 institutions and community contacts and presented to potential participants.

Participants with informed consent and aged between 16 and 25 years were included, and parental consent was obtained if participants were recruited from community organizations and were below 18 years old. Using unique links, data was collected to objectively identify the recruitment source for each participant. The yield per strategy and characteristics of participants between channels are reported elsewhere [23].

### Procedures

The study was conducted through an OWC platform consisting of a landing Web page and capabilities to manage consent, sign up, randomize, administer study measures at different time points, monitor engagement, and provide feedback to users in a meaningful graphic display. Study advertisements linked participants to the landing page of the OWC which contained a brief overview of the study, detailed participant information sheet, and a Web-based consent form.

After completing the Web-based consent form, participants completed a registration form. The OWC software randomized the participants and sent them their login details, either via email or SMS (short message service). Participants logged into the OWC to complete study measurements and if they were assigned to the intervention arm, a link to the intervention website (The Toolbox) was accessible through the OWC immediately. Participants in the control group accessed “The Toolbox” 4 weeks after registration. During the study period, participants from both the intervention and control arm received weekly SMS or email prompts at a time chosen by them during registration, encouraging them to log in to the OWC. The prompts contained a unique link which when clicked logged them in and took them to a page to complete their EMAs. After completing these assessments, they were directed to the OWC homepage, which contained “The Toolbox” access link for the intervention group, and generic well-being advice for the control group. They also received prompts to log in to the OWC to complete study measures at 4 weeks or until they completed.

### Intervention

**Figure 1:**
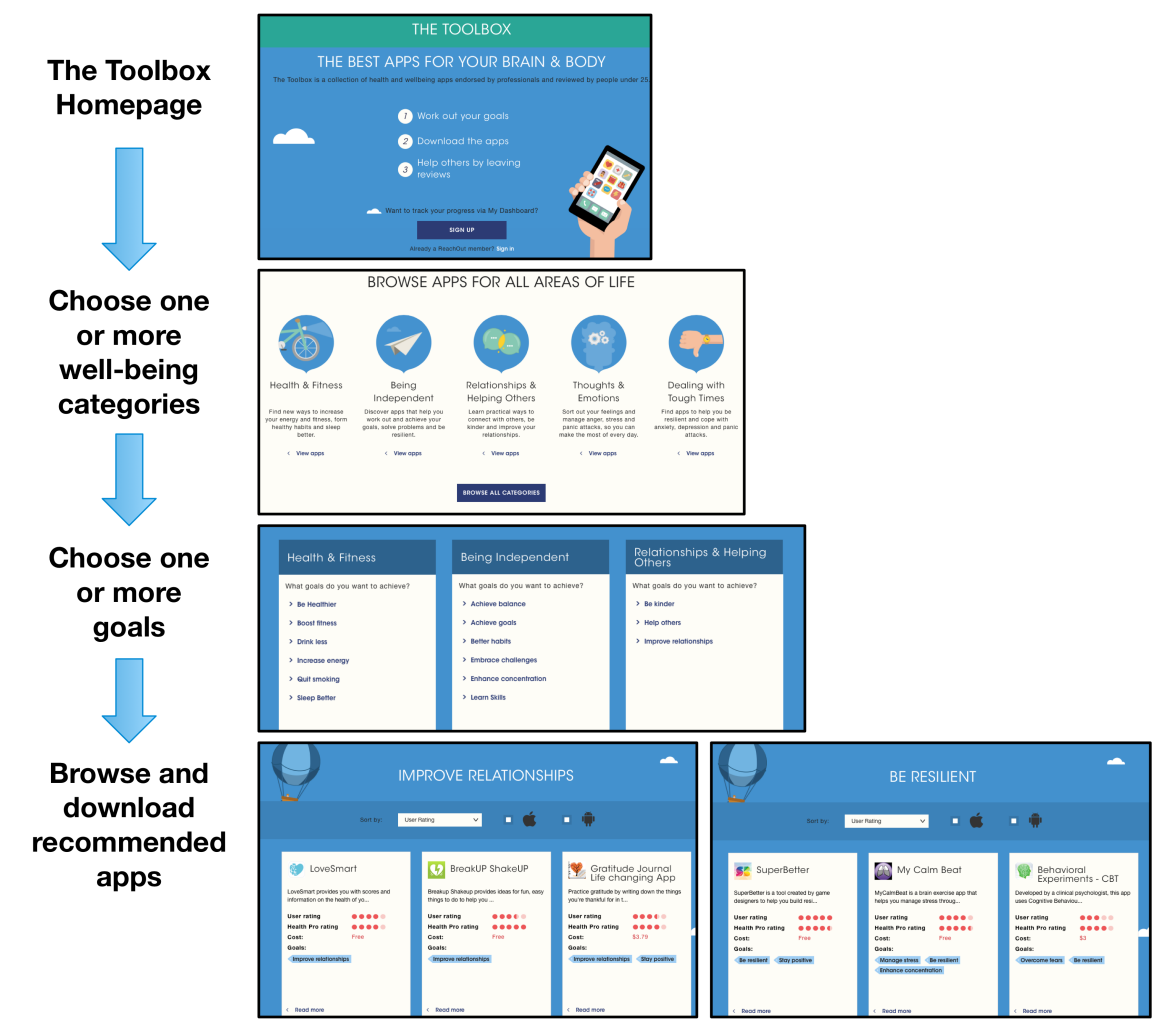
Flow of “The Toolbox” website, including home, well-being category selection, goals selection, and recommended apps pages.

**Figure 2:**
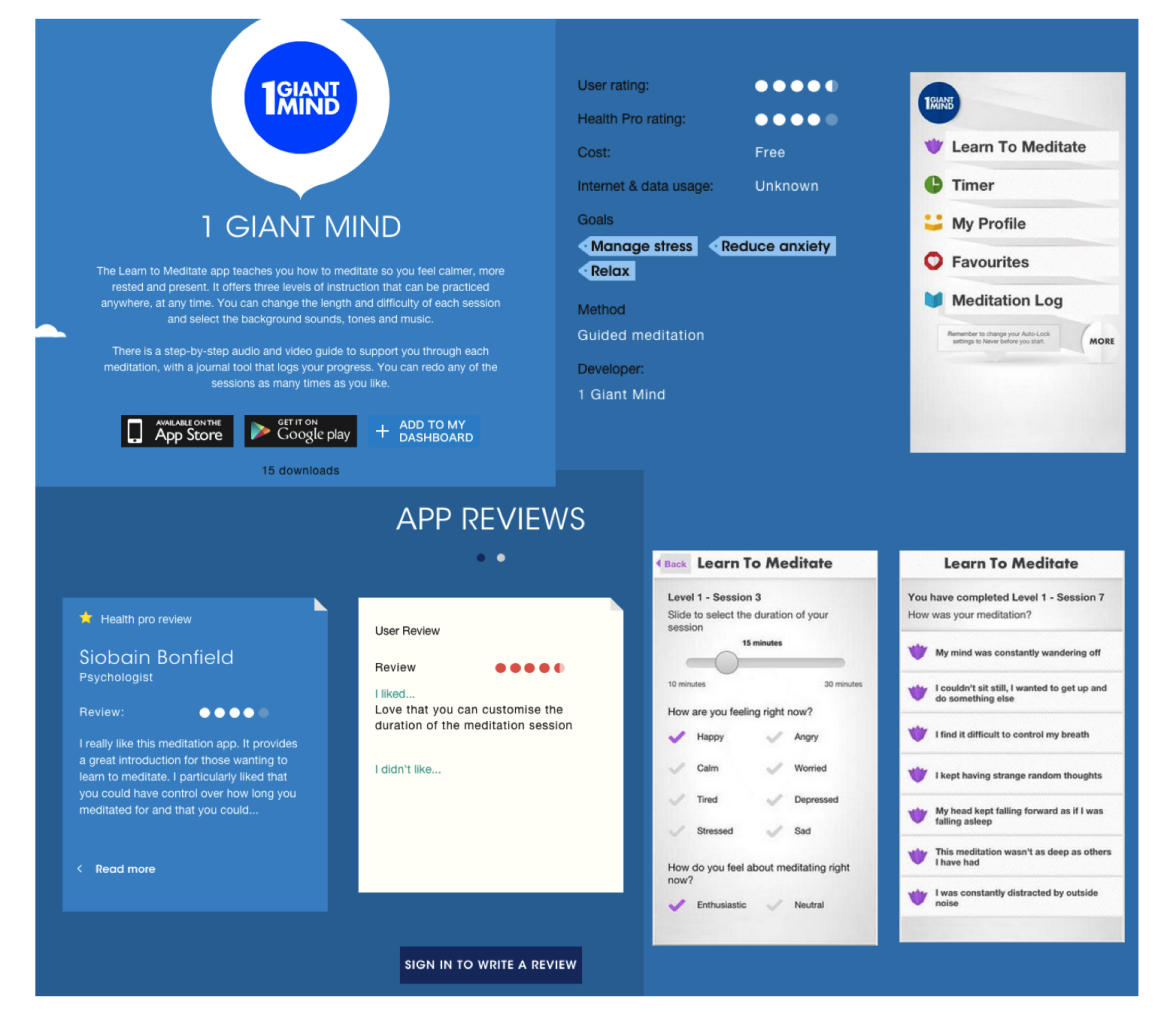
Example of information related to each app available on “The Toolbox” website, including app overview, link to download, user and professional ratings, and app reviews.

The intervention was a personalized app recommendation service called “The Toolbox,” available through the ReachOut.com website [24]. The content and structure of the Web-based intervention was determined by young adults’ perspectives on well-being and expectations with Web-based interventions. End-user studies were conducted with Australian young adults to investigate how young adults conceptualize well-being. Data were collected via user experience workshops with young people aged 15-21 years. Key findings from the workshop influenced the structure and content of the developed Web-based intervention. The workshops with young people were analyzed, resulting in a nuanced understanding of young adults’ conceptualization of health and wellness, and an empirical knowledge of concrete behaviors and actions in their daily lives that they associate with well-being. Data from the workshops were subsequently synthesized into 27 key action areas or goals, and categorized into 6 overarching key themes: health and fitness (15 apps), being independent (8 apps), relationships and helping others (3 apps), thoughts and emotions (18 apps), and dealing with tough times (14 apps). Of these apps, 31 were available for free, 12 apps were paid with costs of up to Aus $6.49, and 3 apps either offered a free lite version or were available for free on one of the platforms, but not on the other. The process of selecting apps to populate “The Toolbox” consisted of two stages. First, a contextual review of available apps was conducted, followed by a review of these apps according to the MARS [10] by professionals and young people. For the contextual review, a list of key search terms was created (see Multimedia Appendix 1), which was drawn from a conceptual well-being model of promoting resilience and flourishing developed for the Young and Well Cooperative Research Centre, as well as qualitative input gained in workshops with young people. These terms were then used to conduct an audit of existing well-being mobile apps available on Google Play and the Apple App Store in late 2013. Only apps that (1) appeared in the first 200 search results, (2) were under Aus $5, (3) were available for Android or iOS, and (4) were deemed appropriate for 13-25 year olds, were included in the rating process. During the rating process, irrelevant apps were removed as well as those not meeting minimum functionality and aesthetic requirements. Remaining apps were rated by researchers using the MARS for both effectiveness and usability and only the highest scoring apps were selected for additional rating by a mental health expert. Apps that contained valid information and were deemed not harmful for young people were selected for “The Toolbox” and additionally rated on the MARS by at least two young people. The final curated list of 46 readily available apps, categorized according to the 27 goals, were put together into a Web resource called “The Toolbox,” with an average of 3.62 (SD 3.05) apps per goal (see Multimedia Appendix 2). “The Toolbox” is a responsive website hosted by Reachout.com. Participants first choose the areas they want to focus on, guided by an interactive quiz and subsequently receive recommendations for particular apps to download and use based on their preferences (see Figure 1). For each recommended app, additional information is provided, including the MARS score and reviews by both health professionals and end users on what they liked and did not like, along with costs and links to download from the app store (see Figure 2).

Participants assigned to the intervention arm upon completion of their baseline measures were displayed a Web link which gave them immediate access to “The Toolbox.” Over the 4-week study period, participants were sent weekly reminders (via email or SMS) advising them to visit “The Toolbox” at least once, take the quiz, and use the recommended apps. The use of “The Toolbox” website and the recommended third-party apps constitutes the intervention in this study. Participants were aware at all times that the researchers assumed no responsibility for the content and/or functionality of these apps.

### Control

Participants in the control group were advised that they were on a waiting list for 4 weeks before they would be given a link to access “The Toolbox.” At 4 weeks after completing the baseline measures, participants were provided access to “The Toolbox” via the OWC.

### Measures

**Figure 3:**
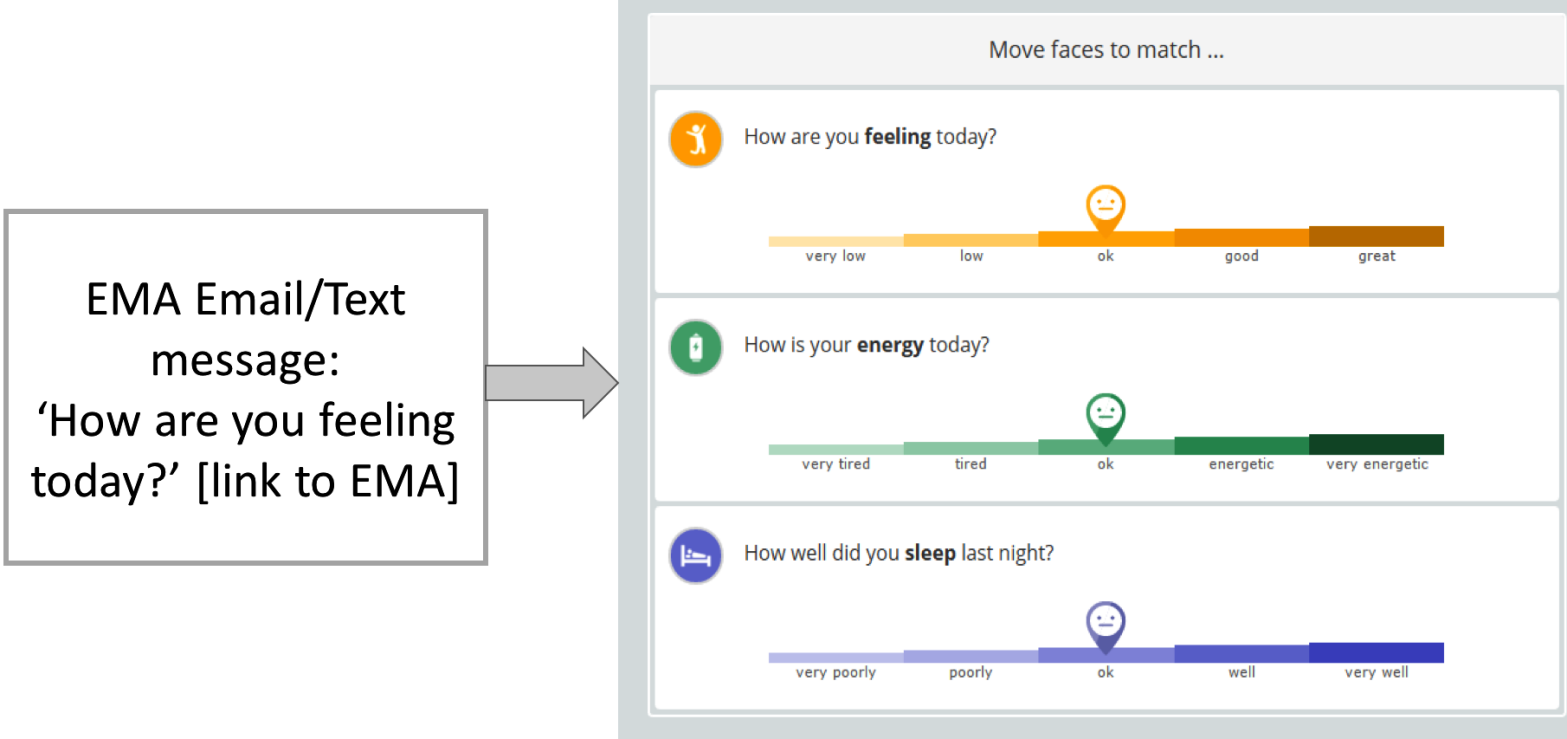
Text message and ecological momentary assessment (EMA).

The primary outcome was a self-reported measure of well-being assessed using the 14-item MHC-SF that measures subjective psychological, emotional, and social well-being. Secondary outcomes were EMAs of 3 questions: How are you feeling today? How is your energy level today? How well did you sleep last night? (see Figure 3). Participants completed primary outcome measures on the Web at baseline and 4 weeks. The EMAs were completed each time participants logged into the OWC during the study period. The log file from the Web app during the trial period was gathered to derive engagement with the study platform. The EMAs of participants were obtained for up to 6 months’ postcompletion of trial.

### Statistical Analysis

Differences in attributes between groups (Table 1) and in attrition versus not (Table 2) were assessed using chi-squared tests, *t* test or Wilcoxon rank-sum tests as appropriate. The primary analysis was based on intention to treat, and missing values from all randomized participants were imputed with 50 samples redrawn from the original data. The primary outcome was analyzed using linear regression (Table 3) with well-being score at 4 weeks as the dependent variable. The independent variables were well-being, measured at baseline, and group assignment. A multivariable intention to treat linear regression sensitivity analysis was also conducted, as well as the same analysis, using observed data only. To investigate how the momentary assessment of mood, sleep, rest, and energy were influenced by the intervention (Table 4), the trajectories of momentary assessment measures were examined using random effects mixed modeling. The independent variables were group assignment, engagement with the study platform (coded as number of logins), the product term of group assignment, and engagement, and potential confounders were age, gender, prior application use (coded as a binary variable), baseline energy, baseline mood, and whether or not an app was downloaded. Subject was entered into the model as a random effect to account for correlated readings within an individual. Differences between groups were assessed using interaction terms. Similar multivariable linear regression analyses were conducted with the MHC-SF subscales as outcomes, with covariates listed as before. To examine whether engagement with the study platform was associated with changes in the EMA measures, a linear regression model was run with postintervention EMA measures as the outcome. All models contained an additional term representing the number of logins. For energy, mood, and rest, the other covariates were listed before in the sensitivity analyses. For sleep the other covariates were baseline sleep and group assignment due to the small number of observations. For these regressions, postintervention measurements for EMAs were taken as the measurement that occurred between 30 and 45 days, with the earliest one after 30 days. All results are reported with 95% CI and *P* values. A *P* value <.05 (2-tailed) was taken to be significant. All analyses were performed using Stata version 13.1 (StataCorp).

## Results

### Flow of Participants

**Figure 4:**
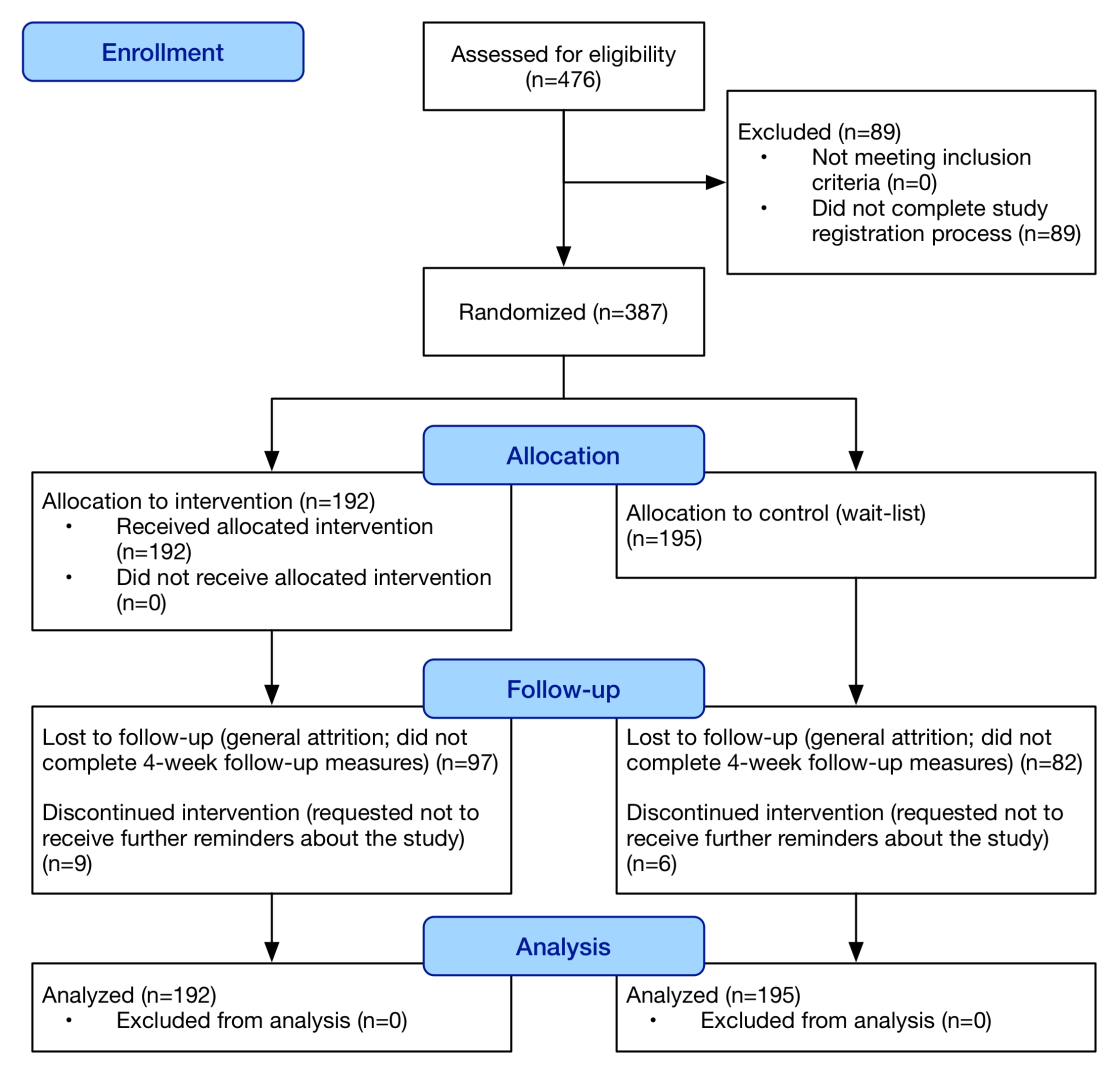
Consolidated Standards of Reporting Trials (CONSORT) flow diagram of study participants during study enrolment, allocation, follow-up, and analysis.

Figure 4 shows the flow of participants. A total of 476 people were consented and signed up on the Web. Of these, 387 completed baseline scores and were randomized into the control (n=195) and active (n=192) arm of the trial.

#### Participant Characteristics

The demographic characteristics and baseline scores are shown in Table 1.

**Table 1:** Baseline characteristics.

Participant characteristics	Control	Intervention	Total	Statistics	*P* value
Age in years, median (interquartile range)	23 (20-25)	23 (20-25)	23 (20-25)	*z*=0.67	.51
Female gender, n (%)	152 (78.4)	143 (75.3)	295 (76.8)	χ^2^_1_=0.5	.47
Prior app usage, n (%)	72 (36.9)	65 (33.9)	137 (35.4)		
MHC-SF^a^, median (interquartile range)	41 (28-51)	39 (27-51)	40 (27.5-51)	*t*_358_=0.16	.44
Subscale: emotional, median (interquartile range)	10 (7-12)	10 (8-12)	10 (7-12)	*z*=0.24	.81
Subscale: social, median (interquartile range)	13 (7-17)	12 (6-17)	12 (6-17)	*z*=0.83	.41
Subscale: psychological, median (interquartile range)	18 (11-22)	18 (11-23)	18 (11-23)	*z*=0.03	.98
EMA^b^ “rest,” median (interquartile range)	50 (36-65)	50 (35-64)	50 (36-54)	*z*=0.03	.98
EMA “mood,” median (interquartile range)	50 (40-70)	50 (40-70)	50 (40-70)	*z*=0.03	.98
EMA “energy,” median (interquartile range)	50 (40-60)	50 (30-60)	50 (40-60)	*z*=0.03	.98

^a^MHC-SF: Mental Health Continuum-Short Form.

^b^EMA: ecological momentary assessment.

### Attrition

Attrition measured as the failure to respond to the primary outcome measurements at 4 weeks postrandomization was 45.1% in the control group (88/195) versus 55.2% in the active group (106/192), *P*=.047. In the control group, the mean baseline MHC-SF for those who responded at 4 weeks, versus those who did not was 40.2 versus 37.6 (*P*=.25). In the intervention group, the mean baseline MHC-SF for those who responded at 4 weeks, versus those who did not was 36.7 versus 42.3 (*P*=.12).

A comparison of characteristics between completers (ie, those that reported data on the primary outcome at 4 weeks) and noncompleters revealed no significant differences across all demographics at baseline, apart from mood as assessed through EMA (Table 2).

**Table 2:** Baseline characteristics for those who provided data at four weeks versus not.

Participant characteristics^a^	Noncompleters	Completers	Statistics	*P* value
Age in years, median (interquartile range)	23 (20-25)	23 (20-25)	*z*=0.05	.96
Female gender, n (%)	14 (77.0)	148 (76.7)	χ^2^_1_=0.0	.95
Prior app usage, n (%)	69 (35.6)	68 (35.2)	*t*_358_=0.50	.94
MHC-SF^a^, median (interquartile range)	41 (27-50)	40 (28-52)	*t*_358_=0.50	.78
Subscale: emotional, median (interquartile range)	10 (7-12)	10 (8-12)	*z*=0.82	.41
Subscale: social, median (interquartile range)	12 (6-17)	12 (7-17)	*z*=0.49	.62
Subscale: psychological, median (interquartile range)	19 (11-22)	18 (12-23)	*z*=0.14	.89
EMA^b^ “rest,” median (interquartile range)	50 (35.5-63)	50 (36-65)	*z*=0.18	.86
EMA “mood,” median (interquartile range)	50 (40-70)	60 (50-70)	*z*=2.13	.03
EMA “energy,” median (interquartile range)	50 (30-60)	50 (40-60)	*z*=0.66	.51
EMA “sleep,” median (interquartile range)	420 (375-480)	435 (360-525)	*z*=0.22	.85

^a^MHC-SF: Mental Health Continuum-Short Form.

^b^EMA: ecological momentary assessment.

### Primary Analysis

The mean (SD) observed MHC-SF scores at 4 weeks for the control and active groups were 38.6 (SD 15.4) and 42.0 (SD 16.8), respectively. In the primary intention-to-treat (ITT) analysis, those in the intervention group experienced an improvement of 0.63 (95% CI −2.26 to 3.53) in MHC-SF score relative to the control group, but this was not significant, *P*=.66. In multivariable sensitivity intention to treat analysis the difference was almost identical, 0.64 (95% CI −2.27 to 3.54), *P*=.66. In a further multivariate sensitivity analysis with observed data only, there was also no difference between groups, 1.10 (95% CI −1.68 to 3.89), *P*=.44. In a completers only analysis there was also no difference between groups in MHC-SF scores 1.17 (95% CI −1.98 to 3.53), *P*=.40.

### Subscales of Mental Health Continuum-Short Form (MHC-SF)

There were no significant differences between groups in any of the subdomain scores, psychological 0.57 (95% CI −0.67 to 1.81), *P*=.95, social 0.46 (95% CI −0.68 to 1.59), *P*=.42, and emotional −0.02 (95% CI −0.72 to 0.68), *P*=.95.

### Analyses of Ecological Momentary Assessments

For all EMA measures, the control group decreased significantly per login in contrast to the active group which showed no significant change in scores over time. Thus, the difference between groups per login was also significant (Table 4).

**Table 3:** Multivariable intention to treat analyses (adjusted for group assignment, age, gender, prior app use, energy, mood, and whether or not an app has been downloaded).

Outcome Measure	Value beta (95% CI)	*P* value
MHC-SF^a^	.64 (−2.27 to 3.54)	.66
Psychological	.57 (−0.67 to 1.81)	.95
Social	.46 (−0.68 to 1.59)	.42
Emotional	−.02 (−0.72 to 0.68)	.95

^a^MHC-SF: Mental Health Continuum-Short Form.

**Table 4:** Changes per login in ecological momentary assessments (adjusted for group assignment, age, gender, prior app use, whether or not an application has been downloaded).

Ecological Momentary Assessments	Control beta (95% CI), *P* value	Intervention beta (95% CI), *P* value	Difference between groups beta (95% CI), *P* value
Mood	−.25 (−0.40 to −0.11)_,_*P*=.001	.15 (−0.04 to 0.33), *P*=.13	.40 (0.16-0.63), *P*=.001
Energy	−.20 (−0.33 to −0.07), *P*=.003	.11 (−0.06 to 0.29), *P*=.19	.31 (0.10-0.52), *P*=.004
Rest	−.19 (−0.36 to −0.08), *P*=.001	.12 (−0.04 to 0.29), *P*=.15	.31 (0.11-0.52), *P*=.002
Sleep	−2.34 (−3.16 to −1.52), *P*<.001	−.46 (−1.66 to 0.74), *P*=.15	1.88 (0.43-3.34), *P*<.001

### Engagement-Response Analysis

There was no evidence to suggest that a beneficial effect was associated with the number of logins in any of the measures, mood 0.15 (95% CI −0.58 to 0.87), *P*=.69, energy 0.08 (95% CI −0.56 to 0.72), *P*=.81, rest −0.13 (95% CI −0.91 to 0.64), *P*=.73, and sleep −5.29 (95% CI −11.96 to 1.54), *P*=.12.

## Discussion

### Principal Findings

The aim of this study was to assess the efficacy of “The Toolbox” Web-based well-being intervention in a young adult population. Results from the randomized controlled trial demonstrated no significant benefit on well-being (as assessed using the MHC-SF) at 4 weeks compared with the control group. There were no significant differences between the active and control groups at 4 weeks on any of the subscales of the MHC-SF either. The trial results also suggest that the impact of receiving weekly texts and the opportunity to monitor and visualize sleep, mood, and energy led to repeated logins in both control and active groups. In addition, participants in the control group reported a significant decline in mood, energy, rest, and sleep as assessed with EMAs with an increasing number of logins, whereas the intervention group showed no change. Thus, repeat engagement with the intervention might halt decline in mood, energy, rest, and sleep, without resulting in significant changes in well-being as assessed by the MHC-SF at fixed points. It remains unclear as to whether this interaction can be attributed to using “The Toolbox” intervention or using the study platform and its repeated assessments. The magnitude of change in the control group was very small, which may explain why no change in well-being as measured by the MHC-SF was observed.

### Comparison With Previous Work

The lack of effect on MHC-SF well-being scores observed in this intervention are comparable with results from similar Web-based intervention trials, conducted in older (mean age 43.2 years) [25] and under 16-years-old school-based samples [26]. Across both studies, positive benefits were shown in depression scales but benefits assessed using mental well-being scales themselves were minuscule and nonsignificant both immediately after the intervention and at follow-up. Compared with these studies, the intervention in our study was targeted at a general young adult sample (mean age 23 years) with a broad inclusion criterion that did not exclude participants based on symptom screening, which closely resembles the real-world setting of intervention delivery through the Reachout.com website. Considerably more females than males participated in this study, which is in concordance with the majority of research into mental health and well-being interventions. In part, the higher proportion may be attributed to the higher prevalence of mental disorders in females in this age group [27]. However, differences in help-seeking behavior between males and females likely account for the majority of this difference [28].

In our study, instead of administering depression scales, we assessed symptoms of depression at multiple time points through momentary assessments. Despite the differences in type of assessment, we detected comparable benefits on depression as evident by significant improvement in mood trajectories in the intervention group. One plausible explanation from these findings is that modest improvements detected in mental well-being might actually be a reduction in depression symptoms that have collinearity with mental well-being [29]. This raises questions about sensitivity of mental well-being scales to detect change and if Web-based interventions can change mental well-being as an independent construct in the absence of mental illness.

Compared with past studies, the intervention in this study is unstructured, in the form of a collection of curated list of mobile app resources accessible through a self-guided hub, as well as monitoring tools to engage participants and provide feedback irrespective of app use. Disseminating a curated list of mental health and well-being apps for depression and anxiety alone has been recently demonstrated to yield better app uptake [13]; however, ours is the first study to investigate effectiveness of such an intervention. In order to ensure optimal app recommendations, different components and strategies within apps that serve as active intervention ingredients must thus be identified [30] and aligned with end user needs. The intervention in this study included a broad range of curated apps (n=46) with an algorithm that assigned these apps to one of the 27 action areas used in the app selection quiz, that were identified based on young adults’ conceptualization of well-being through codesign activities. Since the spread of apps were not uniform across all action areas, the collection of apps might not have been optimal to be suitable and effective for all individuals. We also observed a slightly higher baseline symptomology in participants dropping out from the intervention, which might be caused by the mismatch between apps and individual health circumstances that were not factored in the matching algorithm.

The use of specific apps over a 1-month period may not have been sufficient to induce significant changes in well-being as measured by a global mental health scale, such as the MHC-SF. Instead, our results suggest that app usage may affect momentary moods and behaviors more easily measured by EMAs. It may be that EMAs provide a more accurate measure of the day-to-day impact of app usage. To date, there are few studies of well-being interventions utilizing momentary assessments as outcome measures, although their superiority to traditional questionnaire measures has recently been reported [31].

### Limitations

There are several limitations to our study. There was a high attrition rate of almost 50% subject randomized, which is not unusual in Web-based interventions. However, the results of the primary analysis were consistent with the sensitivity analyses. Interestingly, we found a higher rate of attrition in the intervention group compared with the control group, a finding which was also reported by Bolier et al [25]. The reason for the greater attrition rate in the intervention group is unclear and, along with many factors associated with the high attrition rates in Web-based interventions, requires further study [32]. Given that intervention and study dropout are often linked in Web-based interventions, it is possible that overall attrition was higher in the intervention group because participants immediately had access to the intervention and thus had no incentive to participate in the 4-week assessment. Alternatively, it is possible that the content, functionality, and aesthetics of some apps may have changed during the short time from when they were added to “The Toolbox” to when they were accessed by participants, as is common on mobile app marketplaces [33], thus not meeting, or differing significantly from their expectations. All of the recommended apps remained available on the app stores for the duration of the study.

Due to the nature of the intervention, it was not possible to quantify the download and use of the apps recommended by “The Toolbox.” Thus, there was no direct measure of intervention adherence. This was accounted for by using ITT analysis; however, ultimately it was not possible to determine whether the lack of effect on well-being was due to nonadherence or due to a lack of effectiveness of “The Toolbox” and its recommended apps. This is an inherent problem when studying the effectiveness of third-party intervention that can only be overcome by retaining full ownership of the intervention tool, as was the case in Lattie et al [13]. The study was also limited by a relatively heterogeneous sample of participants recruited using varying strategies, although this could be both a strength and limitation as it replicates app recommendation interventions in real-life setting. The other major limitation was the lack of longitudinal follow-up data; however, it is unlikely longitudinal effects would be found when no benefits were observed at follow-up (4 weeks), which is when most changes are expected.

### Conclusions and Recommendations for Future Research

Whereas there are several randomized controlled trials of the efficacy of Web-based services to improve mental health, previous studies have been conducted in adults with symptoms of anxiety and depression [25,34,35]. In comparison, this was the first study to assess the effectiveness of a well-being intervention designed to recommend the use of readily available mobile apps in a sample of young adults. The design of the intervention utilized expert rating of existing apps and end-user codesign approaches, resulting in an app recommendation service.

Our findings cast doubt on the effectiveness of mobile apps for well-being and mental health in a nonclinical population of young adults. This intervention included a self-guided optimal selection of apps. Further work could focus on developing algorithms to automate the process of determining optimal apps for an individual, taking into account active ingredients in apps, personal characteristics, engagement, and needs. Intervening with the right combination of quality apps is critical to realizing benefits of over 30,000 mental health related apps available in the app store. The instruments used for assessing mental well-being in this study may not have been sensitive enough to detect change caused by app usage. Future research should focus on refining the construct so that it is sensitive to change even when symptoms of depression or mental illness are absent. In addition, consideration should be given to the measurement of the specific behaviors targeted by particular apps as well as to overall constructs, such as well-being.

## Appendix

Multimedia Appendix 1 List of key search terms used to conduct an audit of existing well-being mobile apps available on Google Play and the Apple App Store, during the app selection process for “The Toolbox” intervention.

Multimedia Appendix 2 List of apps contained within “The Toolbox,” classified according to their overarching key themes and action areas or goals.

Multimedia Appendix 3 CONSORT E-HEALTH checklist form.

## Abbreviations

ANZCTR: Australian New Zealand Clinical Trials Registry
BCT: behavior change technique
DECD: Department of Education and Child Development of South Australia
EMA: ecological momentary assessment
ITT: intention-to-treat
MARS: mobile app rating scale
MHC-SF: Mental Health Continuum-Short Form
OWC: Online Wellbeing Centre
SMS: short message service
