# Supplementary material for: Efficacy of a Web-Based Guided Recommendation Service for a Curated List of Readily Available Mental Health and Well-Being Mobile Apps for Young People: Randomized Controlled Trial
Source: J Med Internet Res. 2017 May 12;19(5):e141. doi: 10.2196/jmir.6775 (PMC5446666; doi:10.2196/jmir.6775)
Supplement: Multimedia Appendix 1 [file jmir_v19i5e141_app1.pdf]

TOOLS AUDIT SEARCH TERMS:				
Acceptance	Failing	Managing conflict	Relaxation	Stress
Accomplishment	Family (Note: lots of family history and family tracking apps)	Meeting new people	Satisfaction	Work and career
Achievement	Feeling connected (community)	Mental fitness	Seeking help (no relevant results)	
Achieving a balance in life (life balance)	Fitness	Mood	Self-acceptance	
Altruism	Finding your place (belonging)	Money	Self-belief	
Autonomy	Friendship (Note: lots of friendship tests)	Motivation	Self-esteem	
Caring for others (caring)	Goal setting	New experiences	Self-talk and self-awareness	
Challenging yourself (comfort zone)	Gratitude	Optimism	Sense of purpose (no relevant results)	
Communication *(Note: lots of communication aids and social messaging tools)*	Habits	Partying safe	Setting goals	
Confidence	Happiness	Personal growth	Setting priorities (no relevant results)	
Coping	Healthy	Personal identity	Sex	
Cognitive Behaviour therapy	Hope (Note: search returned lots of religious apps)	Physical health	Sleep	
Dealing with a relationship break-up	Independent (Note: search returned lots of games and Independence day themes)	Planning for the future	Sleeping issues	
Dealing with change	Interest in life (no relevant results)		Socialising (Note: search mainly returned social networking sites)	
Diet	Keeping calm (anxiety)	Positive relations	Social (Note: search mainly returned social networking sites)	
Eating well	Leaving home	Positive self-talk	Spending time with friends	
Empowered (Note: no valid results)	Legal issues	Positivity	Strengths	
Enjoyable activity	Life choices	Problem solving		
Environmental mastery	Looking after yourself	Promoting harmony		

Exams stress	Making a contribution	Purpose in life		
Study stress	Resilience	Relationships		
Exercise				

TOOLS AUDIT "EXCLUSION" TERMS				
Happiness -quotes				
Meditation –yoga				
Positivity –quotes				
Relaxation –hypnosis -yoga				
Confidence –quotes<br>-hypnosis				
Friendship -quotes				
