# Supplementary material for: Efficacy of a Web-Based Guided Recommendation Service for a Curated List of Readily Available Mental Health and Well-Being Mobile Apps for Young People: Randomized Controlled Trial
Source: J Med Internet Res. 2017 May 12;19(5):e141. doi: 10.2196/jmir.6775 (PMC5446666; doi:10.2196/jmir.6775)
Supplement: Multimedia Appendix 2 [file jmir_v19i5e141_app2.pdf]

	1 Health and fitness						2 Being independent						3 Relationships and helping others			4 Thoughts and emotions							5 Dealing with tough times				
**Apps / Goals**	Be healthier	Boost fitness	Drink less	Increase energy	Quit smoking	Sleep better	Achieve balance	Achieve goals	Better habits	Embrace challenges	Enhance concentration	Learn skills	be kinder	help others	improve relationships	improve general wellbeing	increase happiness	manage anger	manage stress	overcome fears	Relax	Stay Positive	Be resilient	control panic attacks	manage depression	manage anxiety	manage eating disorders
1 Giant Mind																			X		X					X	
101 Revolutionary ways to be healthy	X	X																									
365 Happy Days																X	X										
8 glasses a day	X																										
Argus		X																									
Balanced								X																			
Beat panic																								X			
Behavioural Experiments - CBT																				X			X				
Bodywise Health and Fitness Tracker	X	X																									
Breathe 2 relax																		X	X							X	
bupa fitness		X																									
Calm meditations and relaxation						X															X						
Couch to 5k	X	X																									
Dormio						X																					
eCBT Mood																									X		
Everest								X																			
Goal Setter								X				X															
Good Habit Maker								X								X											
Gratitude Journal Life Changing App															X							X					
health tap	X																										
instant heart rate		X																									
Juice	X			X																							
Kindness Meditation													X									X					
learn panic CBT																								X			
mindfulness meditation																			X								
Moodkit																						X					
My Calm Beat											X								X				X				
My quit buddy					X																						
Nike running		X																									
Optimism																									X		
Panic attack aid lite																								X			
Pay it forward													X	X								X					
Proof								X	X	X																	
Relax with Andrew Johnson Lite																			X								
Relaxation Techniques - Calm your mind																			X								
Rise up overcome your eating disorder																											X
Self-Help for Anxiety Management																			X							X	
Smiling Mind																X			X		X	X					
Smoke free					X																						
Spire								X																			
Stop panic and anxiety																								X		X	
super better																						X	X				
Talking anxiety																										X	
The sorter								X																			
Walk Watch Lite	X	X																									
WebMD																X											
